# NS-018 reduces myeloma cell proliferation and suppresses osteolysis through inhibition of the JAK2 and Src signaling pathways

**DOI:** 10.1038/s41408-018-0098-z

**Published:** 2018-06-25

**Authors:** Ayumi Honda, Kazuya Kuramoto, Tomoko Niwa, Haruna Naito

**Affiliations:** Discovery Research Laboratories, Nippon Shinyaku Co., Ltd., Kyoto, Japan

Dear Editor,

Multiple myeloma (MM) is a clonal B-cell malignancy characterized by the infiltration of malignant plasma cells into the bone marrow, and it eventually leads to impaired hematopoiesis, the production of high levels of monoclonal immunoglobulin, and osteolytic bone destruction^1^. Bone pain and compression fractures caused by bone lesions not only severely impair the quality of life of MM patients, but also are associated with an ∼20% increase in the risk of death^2^. The pathophysiology of MM is closely linked with the bone marrow microenvironment. Bone marrow stromal cells release cytokines, such as interleukin-6 (IL-6) which promote the proliferation and survival of myeloma cells^1,3^, whereas myeloma cells secrete osteoclast differentiation factors, such as receptor activator of nuclear factor-kappa B ligand (RANKL), causing osteolysis^4^.

NS-018 is a potent, ATP-competitive small-molecule inhibitor of Janus kinase 2 (JAK2) and Src, which is under development for the treatment of myeloproliferative neoplasm^5,6^. In the present study, we investigated the inhibitory effect of NS-018 on the IL-6/JAK2/STAT3 (signal transducer and activator of transcription) and Src signaling pathways in myeloma cell lines and its biological effects in a mouse model of MM.

**Fig. 1:**
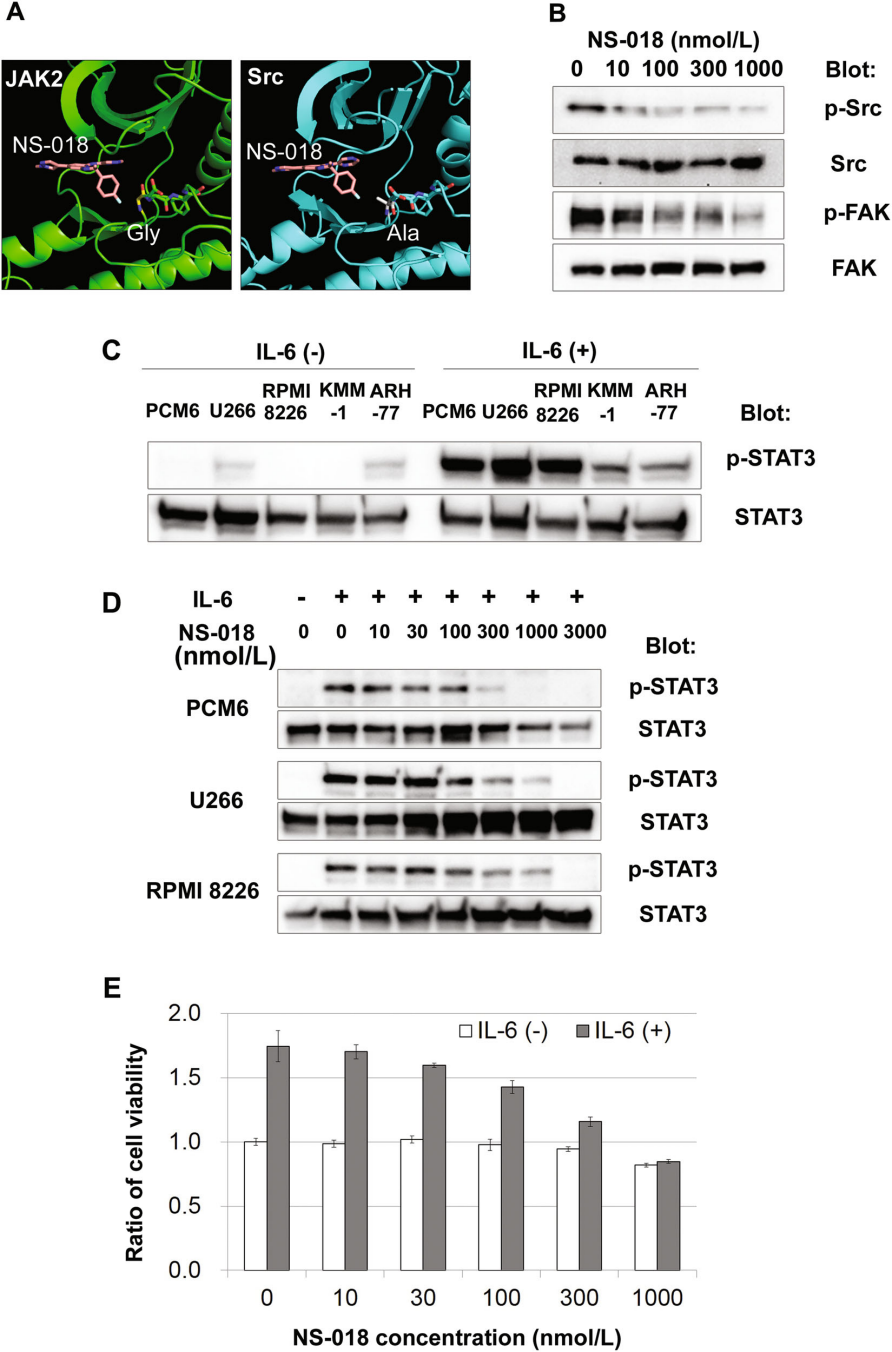
NS-018 suppresses IL-6-induced STAT3 phosphorylation and the proliferation of myeloma cell lines. **a** Docking models. The backbone of JAK2 is shown in green (left panel), the backbone of Src kinase in cyan (right panel), and NS-018 in pink (both panels). For clarity, only the amino acid side chains of the DFG motif are shown. **b** NIH3T3/v-Src cells were incubated with the indicated concentrations of NS-018 for 30 min. Cells were harvested and lysed with cell lysis buffer and the lysates analyzed by western blotting. **c** Myeloma cells were incubated with or without 5 ng/mL IL-6 for 2 h. The cells were harvested and lysed and the lysates analyzed for STAT3 and p-STAT3 by western blotting. **d** PCM6, U266, and RPMI 8226 cells were incubated with 10 ng/mL IL-6 for 2 h and then treated with the indicated concentrations of NS-018 for 30 min. The cells were harvested and analyzed for STAT3 and p-STAT3 as described above. **e** PCM6 cells were incubated with or without 10 ng/mL IL-6 and the indicated concentrations of NS-018 for 3 days and cell viability was analyzed by MTT assay

We previously reported that NS-018 selectively inhibits JAK2 and Src-family kinases in in vitro kinase assays^5^. In the present study, we explored the mechanism of this inhibition in terms of molecular interactions at the atomic level. We began by comparing the modes of binding of NS-018 to Src kinase and JAK2 (Fig. 1a). An in silico study of the docking of NS-018 into the X-ray crystallographic structure of Src kinase showed NS-018 in close proximity to the Ala residue immediately N-terminal to the Asp-Phe-Gly (DFG) motif in the activation loop (Fig. 1a, right panel). In JAK2, the position corresponding to this Ala is occupied by a Gly residue, which is important in the binding of NS-018 to JAK2 (ref.^5^ Fig. 1a, left panel). The presence of a small residue at this position (Gly in JAK2 and Ala in Src kinase) allows the binding of NS-018 to both of these kinases. To test the inhibitory effects of NS-018 on Src signaling at the cellular level, NIH3T3 cells overexpressing v-Src were incubated with NS-018 and the phosphorylation of Src and its downstream effector focal adhesion kinase (FAK) were analyzed by western blotting. NS-018 suppressed the phosphorylation of Src at concentrations of 10 nmol/L or more and of FAK at 100 nmol/L or more (Fig. 1b).

IL-6 is one of the most widely studied cytokines that is elevated in MM patients, and it plays a critical role in the proliferation and survival of myeloma cells^1,3^. Because the JAK/STAT pathway is the major downstream pathway of IL-6 signaling, JAK inhibitors such as ICNB16562, CYT387, and AZD1480 inhibit IL-6-induced MM cell survival^7–9^. To investigate the inhibitory effect of NS-018 on the JAK/STAT signaling pathway, we investigated whether NS-018 inhibited the IL-6-induced proliferation and survival of myeloma cells. First, to examine the responsiveness of MM or plasma cell leukemia cell lines to IL-6, we treated PCM6, U266, RPMI 8226, KMM-1, and ARH-77 cells with IL-6, and analyzed the phosphorylation status of STAT3 by western blotting. In the absence of IL-6, STAT3 phosphorylation was undetectable or barely detectable in all cell lines (Fig. 1c). In contrast, in the presence of IL-6, STAT3 phosphorylation was readily detectable in all cell lines, and the level of phosphorylation was especially high in PCM6, U266, and RPMI 8226 cells, indicating that all cell lines retained IL-6-responsiveness (Fig. 1c). We next investigated the effect of NS-018 on the IL-6-induced phosphorylation of STAT3 in PCM6, U266, and RPMI 8226 cells. NS-018 inhibited IL-6-induced STAT3 phosphorylation in all the three cell lines at concentrations of more than 300 nmol/L (Fig. 1d). To test the effect of NS-018 on IL-6-stimulated myeloma cell proliferation, PCM6 cells, whose proliferation is strongly enhanced by IL-6, were treated with NS-018 in the presence or absence of IL-6. NS-018 inhibited IL-6-induced cell proliferation, but not IL-6-independent cell proliferation, in a dose-dependent manner (Fig. 1e) with an IC_50_ value of 140 nmol/L. These results indicate that the IL-6-dependent growth of myeloma cells was sensitive to NS-018.

**Fig. 2:**
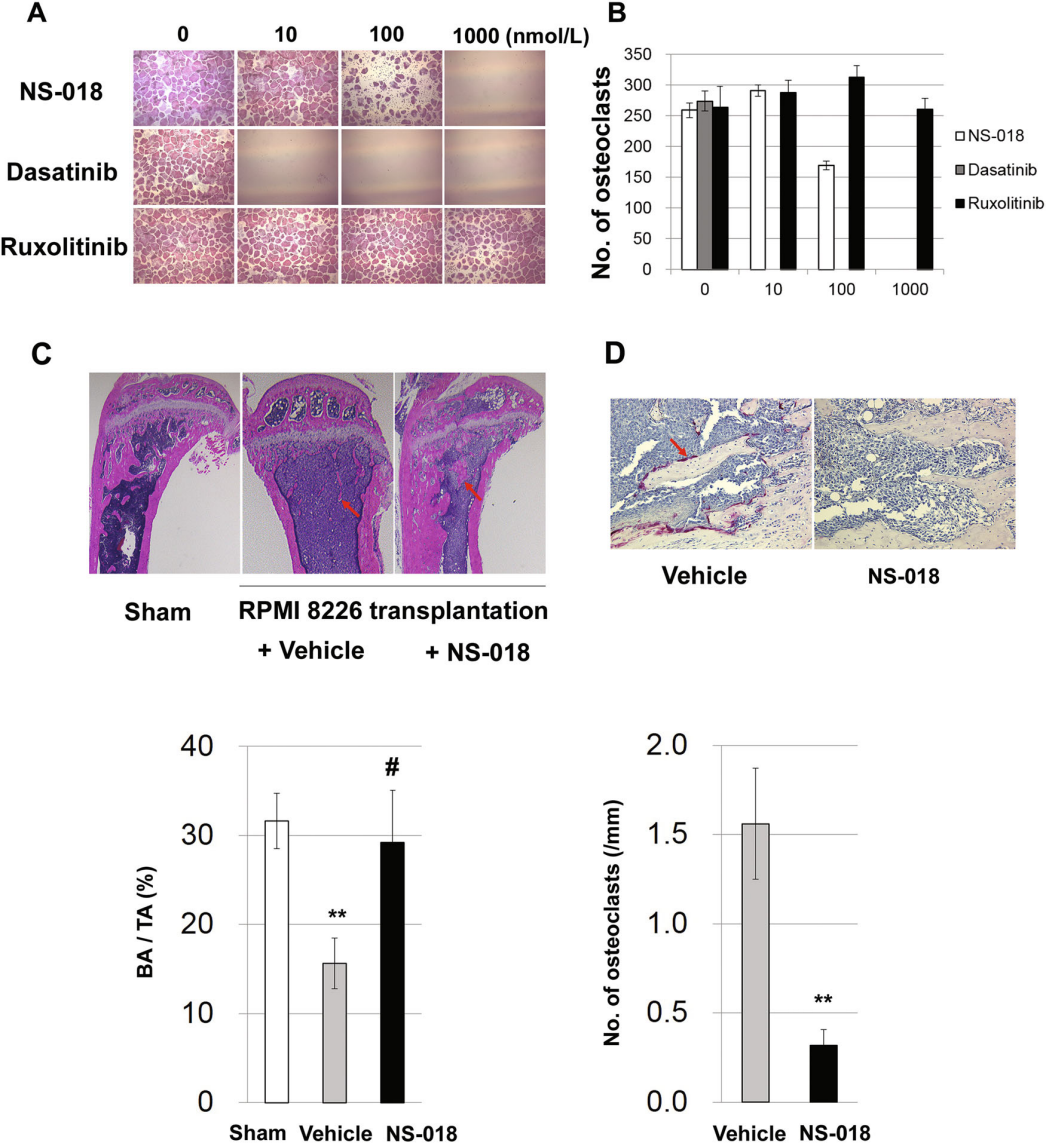
NS-018 inhibits RANKL-induced osteoclast formation and suppresses osteolysis in mice with intratibial transplantation of RPMI 8226 cells. Human osteoclast precursor cells were treated with differentiation-inducing medium containing the indicated concentrations of NS-018, dasatinib, or ruxolitinib for 7 days and TRAP-positive osteoclasts were counted. **a** Representative micrographs. Original magnification ×100 for all panels. **b** Quantification of osteoclasts. Bars represent the mean ± S.E.M. (*n* = 5). **c** Hematoxylin–eosin-stained tibial sections from Sham mice (left micrograph) and myeloma-bearing mice (middle micrograph, vehicle-treated; right micrograph, treated with 50 mg/kg b.i.d. NS-018). Arrows indicate trabecular bone. Original magnification ×40. In the graph showing the trabecular bone area as a percentage of the total tissue area, bars represent the mean ± S.E.M. (Sham, *n* = 15; vehicle or NS-018 treated, *n* = 11). Statistical significance was assessed by Student’s *t*-test (**p < 0.01 vs. Sham; ^#^*p* < 0.05 vs. vehicle-treated). **d** TRAP-stained section of tibia from myeloma-bearing mice (left micrograph, vehicle-treated; right micrograph, treated with 50 mg/kg b.i.d. NS-018). The arrow indicates a TRAP-stained osteoclast. Original magnification ×200. In the graph showing the numbers of osteoclasts per millimeter lining the bone surface, bars represent the mean ± S.E.M. (vehicle-treated, *n* = 10; NS-018-treated, *n* = 9). Statistical significance was assessed by Student’s *t*-test (**p < 0.01 vs. vehicle-treated)

RANKL is a cytokine that is essential for the formation and activation of osteoclasts, and it binds to the RANK receptor expressed on the surface of osteoclast precursor cells. The aberrant production and activation of osteoclasts caused by the dysregulation of RANKL signaling lead to osteoporosis and bone destruction in myeloma patients. Src kinase plays important roles in osteoclast formation by mediating RANKL pathways^4^. Previous studies suggest that Src kinase inhibitors, such as dasatinib and saracatinib inhibit the differentiation of precursor cells into osteoclasts and promote the osteogenic differentiation of mesenchymal stromal cells in vitro and bone formation in vivo^10–12^. In view of the important roles that Src kinases play in the differentiation of precursor cells into osteoclasts, we set out to clarify the contribution of Src inhibition to this process by evaluating the effect of NS-018 on osteoclast formation and comparing it to that of dasatinib, a Bcr-Abl/Src inhibitor, and ruxolitinib, a selective JAK inhibitor. Human osteoclast precursor cells were seeded and induced to differentiate with soluble RANKL and macrophage colony-stimulating factor (M-CSF) in the presence of NS-018 or other kinase inhibitors. After 7 days of culture, TRAP-positive multinucleated mature osteoclasts were counted. NS-018 significantly reduced the number of osteoclasts at a concentration of 100 nmol/L and dasatinib completely suppressed the formation of osteoclasts at 10 nmol/L, whereas ruxolitinib had no effect at any concentration up to 1000 nmol/L (Fig. 2a). Taken together, these results provide evidence that NS-018 inhibited osteoclast formation by the inhibition of Src signaling, but not by the inhibition of JAK/STAT signaling.

To investigate the effects of NS-018 on MM-induced osteolysis in vivo, we injected RPMI 8226 cells directly into the tibial cavity of CB-17 SCID mice. Mice so treated develop trabecular bone loss 4–6 weeks after transplantation^13^. We administered vehicle or NS-018 at a dosage of 100 mg/kg/day for 5 weeks starting the day after transplantation and then sacrificed the mice to evaluate osteolysis. Because no weight loss and no change in general health as determined by a variety of other indicators were observed, it was concluded that a dosage of 100 mg/kg NS-018 was tolerated in this model mouse. Observation of Hematoxylin–eosin (HE) stained specimens from vehicle-treated mice revealed that RPMI 8226 cells had implanted; they could be recognized by their enlarged nuclei frequently containing mitotic chromosomes, and the network structure of the trabecular bone immediately adjacent to the growth plate had drastically regressed (Fig. 2c, middle panel). In NS-018-treated mice, the structure of the trabecular bone was maintained (Fig. 2c, right panel). When the trabecular bone area was measured as a percentage of the total tissue area, there was significantly less loss of trabecular bone in NS-018-treated than in vehicle-treated mice (Fig. 2c). However, we did not observe significant regression of the growth and invasion of RPMI 8226 cells in the tibial marrow cavity in the NS-018-treated mice, so that the effect of NS-018 in suppressing bone loss was not due to the destruction of tumor cells by direct cytotoxicity. To assess the effect of NS-018 on myeloma-induced osteoclast activation (see Fig. 2a), a bone specimen was stained for TRAP (Fig. 2d). In the tibia of vehicle-treated mice, many TRAP-positive osteoclasts were observed on the trabecular and cortical bone surfaces in contact with RPMI 8226 cells, whereas there were few TRAP-positive osteoclasts in the tibia of NS-018-treated mice. There were 80% fewer TRAP-positive osteoclasts per millimeter of bone surface in NS-018-treated mice than in vehicle-treated mice. Taken together, our results provide strong evidence that NS-018 suppressed myeloma-induced trabecular bone loss by suppressing osteoclast activation. RPMI 8226 cells harbor many genetic mutations, such as those in KRAS, p53, and NIK, which activate proliferative and anti-apoptotic signaling pathways^14,15^. For this reason, the proliferation of RPMI 8226 cells is not solely dependent on IL-6, and accordingly the anti-proliferative activity of NS-018 against RPMI 8226 cells was weaker than against PCM6 cells (data not shown).

The result of the osteoclast-formation assay in vitro indicates that inhibition of the Src kinase signaling pathway is predominant in the effect of NS-018 on myeloma-induced osteolysis in vivo. Heusschen and Muller^12^ found that the Src inhibitor saracatinib limits the development of osteolysis in a murine osteolytic model induced by murine myeloma cells. As far as we know, however, the present study is the first demonstration of the use of a Src kinase inhibitor to improve focal osteolysis induced by *human* myeloma cells. This result supports the idea that inhibition of Src kinase could be a therapeutic strategy for MM complicated by bone loss.

In conclusion, NS-018, a novel inhibitor of JAK2 and Src, suppressed not only the IL-6-induced proliferation of myeloma cells but also the development of myeloma-induced focal osteolysis. In addition, NS-018 inhibited osteoclast formation in vitro and in vivo, probably by inhibiting Src kinase. Treatment with NS-018 is a potential new therapeutic option to improve the complex pathological condition of patients with MM.
